# Preliminary population-based incidence and prevalence estimates of systemic lupus erythematosus: the California Lupus Surveillance Project

**DOI:** 10.1186/ar4654

**Published:** 2014-09-18

**Authors:** Maria C Dall'Era, Kurt Snipes, Miriam Cisternas, Caroline Gordon, Charles G Helmick

**Affiliations:** 1 University of California, San Francisco, CA, USA; 2 California Department of Public Health, Sacramento, CA, USA; 3 MGC Data Services, Carlsbad, CA, USA; 4 University of Birmingham, Birmingham, UK; 5 Centers for Disease Control and Prevention, Atlanta, GA, USA

## Background

Previous estimates of prevalence and incidence of systemic lupus erythematosus (SLE) in the United States have varied widely due to factors such as heterogeneous source populations, limitations with case ascertainment, and differing case definitions. The California Lupus Surveillance Project (CLSP) is part of a national effort funded by the Centers for Disease Control and Prevention to determine more credible estimates of incidence and prevalence of SLE, with a special focus on Hispanics and Asians.

## Methods

The CLSP is a population-based registry designed to determine the incidence and prevalence of SLE in San Francisco County, CA, USA. Sources of cases included hospitals, rheumatologists, nephrologists, commercial laboratories, and state population databases. These sources were queried for the International Classification of Diseases, Ninth Revision (ICD-9-CM) codes of 710.0 (SLE), 695.4 (discoid lupus), 710.8 (other specified connective tissue disease), and 710.9 (unspecified connective tissue disease). Laboratories were queried for serologic tests including ANA, anti-dsDNA, anti-Smith, antiphospholipid antibodies, and low complement levels. Pathology laboratories were queried for renal and cutaneous biopsies consistent with lupus. Over 15,000 potential SLE patients were identified after the initial queries, and trained abstractors performed detailed medical chart reviews on the >5,500 patients who met the catchment criteria of residence in San Francisco County within the years 2007 to 2009. Cases were defined as patients with documentation of ≥4/11 of the ACR Classification Criteria for SLE. Using SAS 9.3, we calculated prevalence and incidence rates and associated 95% confidence intervals (CIs). Denominators for all rates were obtained from the US Census data (revised 2000 to 2009 intercensal population files) for San Francisco County.

## Results

The preliminary overall crude prevalence and incidence of SLE in San Francisco County was 90.4/100,000 and 5.1/100,000 respectively. The highest prevalence of disease was observed in Black women (430.6/100,000), followed by Hispanic and Asian (163.8/100,000 and 158.9/100,000, respectively), and White (111.3/100,000) women (Table 1).

**Table 1:** Preliminary prevalence and incidence rates (per 100,000) of SLE in San Francisco County, CA, USA

	Prevalence (2007)		Incidence (2007 to 2009)	
	
Race/ethnicity, sex	Number of cases	Crude rate (95% CI)	Number of cases	Crude rate (95% CI)
Overall	704	90.4 (84.0 to 97.3)	121	5.1 (4.3 to 6.1)
Women	623	162.0 (149.8 to 175.2)	112	9.6 (8.0 to 11.5)
Men	81	20.6 (16.5 to 25.5)	9	0.7 (0.4 to 1.4)
Black	138	243.0 (205.7 to 287.0)	27	15.9 (10.9 to 23.1)
Women	121	430.6 (360.5 to 514.2)	25	29.9 (20.3 to 44.2)
Men	17	59.2 (37.0 to 94.9)	2	2.3 (0.6 to 8.4)
White	255	58.1 (51.4 to 65.7)	43	3.2 (2.4 to 4.3)
Women	230	111.3 (97.8 to 126.6)	38	6.0 (4.4 to 8.3)
Men	25	10.8 (7.3 to 15.9)	5	0.7 (0.3 to 1.7)
Asian	264	95.8 (84.9 to 108.1)	39	4.6 (3.4 to 6.3)
Women	233	158.9 (139.8 to 180.7)	37	8.3 (6.0 to 11.4)
Men	31	24.0 (16.9 to 34.1)	2	0.5 (0.1 to 1.9)
Hispanic	99	87.7 (72.1 to 106.8)	17	4.9 (3.1 to 7.8)
Women	87	163.8 (132.9 to 202.0)	16	9.8 (6.0 to 15.9)
Men	12	20.1 (11.5 to 35.1)	1	0.5 (0.1 to 3.1)

## Conclusions

The CLSP uses more complete case finding methods to provide current estimates of prevalence and incidence in a racially and ethnically diverse population. Racial and ethnic disparities in SLE were confirmed with the highest burden of disease in Black women, followed by Hispanic and Asians, and, finally, White women.

